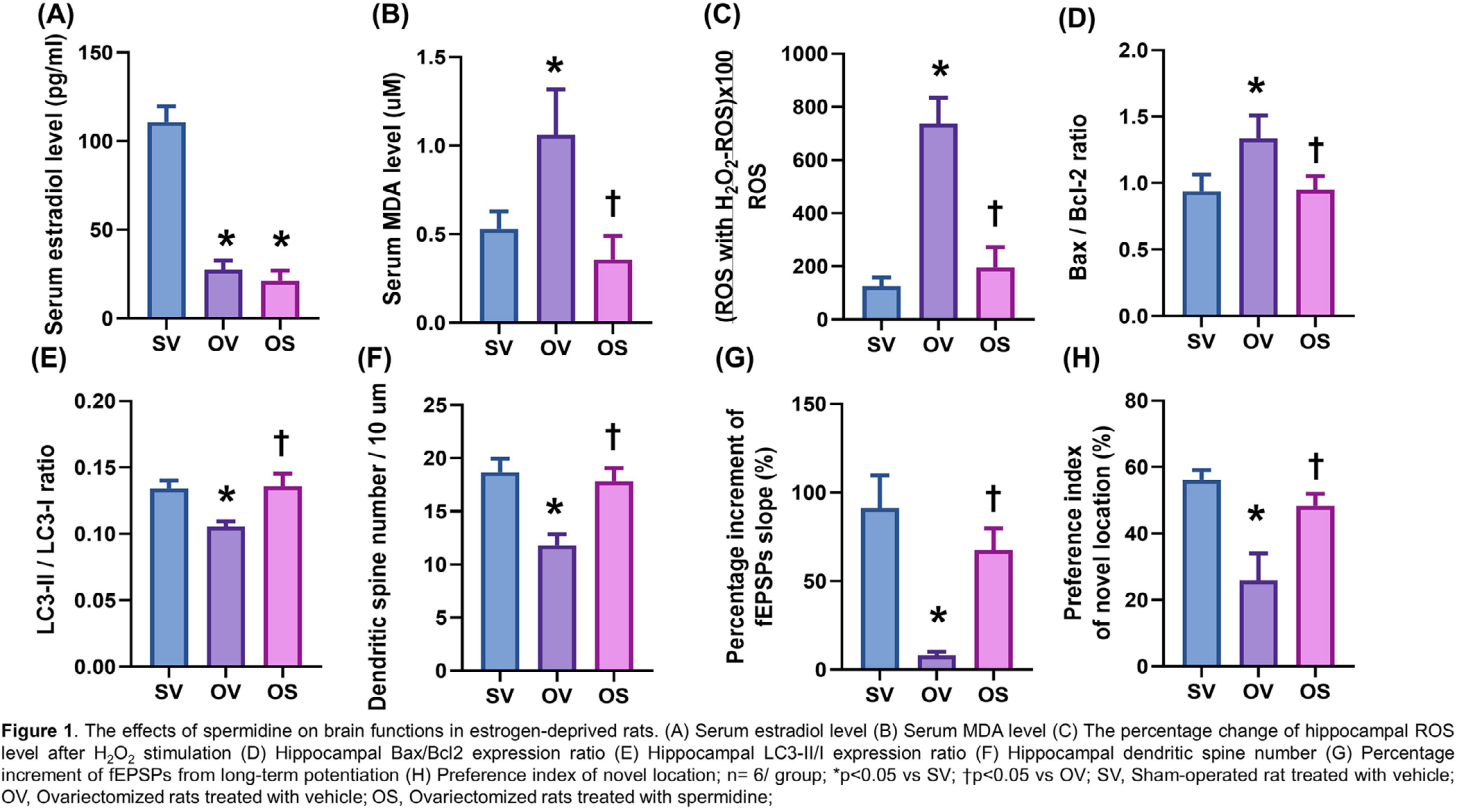# Spermidine Attenuated Brain pathology and Learning Deficit in Estrogen‐deprived Condition

**DOI:** 10.1002/alz.084861

**Published:** 2025-01-03

**Authors:** Wasana Pratchayasakul, Busarin Arunsak, Wichida Kaorop, Chayadom Maneechote, Suriphan Donchada, Aphisek Kongkaew, Titikorn Chunchai, Nipon Chattipakorn, Siriporn C Chattipakorn

**Affiliations:** ^1^ Neurophysiology Unit, Cardiac Electrophysiology Research and Training Center, Faculty of Medicine, Chiang Mai University, Chiang Mai Thailand; ^2^ Cardiac Electrophysiology Unit, Department of Physiology, Faculty of Medicine, Chiang Mai University, Chiang Mai Thailand; ^3^ Center of Excellence in Cardiac Electrophysiology Research, Chiang Mai University, Chiang Mai Thailand; ^4^ Neurophysiology unit, Cardiac Electrophysiology Research and Training Center, Faculty of Medicine, Chiang Mai University, Chiang Mai Thailand; ^5^ Cardiac Electrophysiology unit, Department of Physiology, Faculty of Medicine, Chiang Mai University, Chiang Mai Thailand; ^6^ Cardiac Electrophysiology Research and Training Center, Faculty of Medicine, Chiang Mai University, Chiang Mai Thailand; ^7^ Research Administration Section, Faculty of Medicine, Chiang Mai University, Chiang Mai Thailand; ^8^ Chiang Mai University/Department of Oral Biology and Diagnostic Sciences/Faculty of Dentistry, Chiang Mai Thailand

## Abstract

**Background:**

An increase in the development of learning deficit occurred during estrogen‐deprived periods via the increment of systemic and brain oxidative stress, brain apoptosis, and synaptic dysplasticity. Although estrogen supplementation has been shown to improve the brain function in estrogen‐deprived conditions, it can lead to several adverse effects. Therefore, the novel therapeutic approach with minimal side effects to protect brain function in estrogen‐deprived conditions should be further investigated. Spermidine is a natural polyamine, which can be obtained orally from diet. In the brain, spermidine attenuated age‐induced memory impairment and age‐related locomotor activity loss via the modulation of autophagic process. However, the effects of spermidine on brain function in estrogen‐deprived conditions have never been investigated.

**Method:**

Eighteen female rats were assigned to sham‐operated (Sham; n = 6), or estrogen‐deprived group by ovariectomy (OVX; n = 12). Twelve weeks after surgery period, sham was received vehicle (normal saline; SV; n = 6) for additional 8 weeks. OVX groups were divided into two subgroups to receive either vehicle (normal saline; OV; n = 6) or spermidine (20 mg/kg/day; OS; n = 6) for an additional 8 weeks. At the end of the experimental period, all animals were taken to test the learning process with a novel objective location test. After that, blood and brain were collected to determine systemic and brain parameters.

**Result:**

Ovariectomized rats showed the characteristic of estrogen deprivation, as indicated by decreased estradiol level when compared with those of sham rats (p<0.05, **Figure 1**). Spermidine did not increase the estradiol level of ovariectomized rats. Furthermore, systemic oxidative stress, hippocampal ROS production, hippocampal apoptosis, hippocampal autophagic imbalance, dendritic spine loss, synaptic dysplasticity, and learning deficit were observed in ovariectomized rats (p<0.05, **Figure 1**). Interestingly, spermidine attenuated these systemic and brain pathologies, which lead to improved learning process in ovariectomized rats (p<0.05, **Figure 1**).

**Conclusion:**

These findings suggest that spermidine may be another therapeutic approach for improving systemic and brain functions in the case of estrogen deprivation.